# Case Report: Anterior Scleritis Presenting as a Primary Ocular Manifestation in Multisystem Inflammatory Syndrome in Children With COVID-19

**DOI:** 10.3389/fped.2022.943652

**Published:** 2022-06-27

**Authors:** Daisuke Matsubara, Daisuke Tamura, Yuka Kasuya, Yoshitaka Mizobe, Mami Hiwada, Mitsuru Seki, Shinji Makino, Takanori Yamagata

**Affiliations:** ^1^Department of Pediatrics, Jichi Medical University, Shimotsuke, Japan; ^2^Department of Ophthalmology, Jichi Medical University, Shimotsuke, Japan

**Keywords:** multisystem inflammatory syndrome in children, COVID-19, scleritis, Kawasaki disease, conjunctivitis, fundus findings, ophthalmological examinations, case report

## Abstract

Multisystem inflammatory syndrome in children (MIS-C) is a newly defined hyperinflammatory disease linked to antecedent coronavirus disease 2019. Patients with MIS-C present with various symptoms, and ocular findings such as mild bilateral conjunctivitis are relatively common. However, detailed descriptions of severe ocular reports associated with MIS-C are scarce in the current literature. Here we report a case of MIS-C in a Japanese boy, with severe eye manifestations in the form of anterior scleritis as the primary MIS-C symptom. Detailed ocular examinations by ophthalmologists may be key for clarifying the pathophysiology of MIS-C.

## Introduction

Multisystem inflammatory syndrome in children (MIS-C) has emerged as a new hyperinflammatory disease linked to antecedent coronavirus disease 2019 (COVID-19) (1). MIS-C shares some clinical overlap with Kawasaki disease (KD), a systemic vasculitis, which is common in infants and young children (2). Although it is unclear whether MIS-C and KD are different syndromes or represent a common disease spectrum, both diseases manifest symptoms associated with systemic vascular involvement in various organs, such as the eye, skin, heart (including coronary arterial abnormalities), liver, and kidneys (2). Patients with MIS-C present with various symptoms among which ocular findings are relatively common, such as mild bilateral conjunctivitis (55%) (2). However, detailed descriptions of severe ocular reports associated with MIS-C are scarce in the current literature. Here we report a case of MIS-C in a Japanese boy with severe eye manifestations in the form of anterior scleritis as the primary symptom of MIS-C. The present case partially illustrates the pathophysiology of MIS-C.

## Case Description

**Figure 1:**
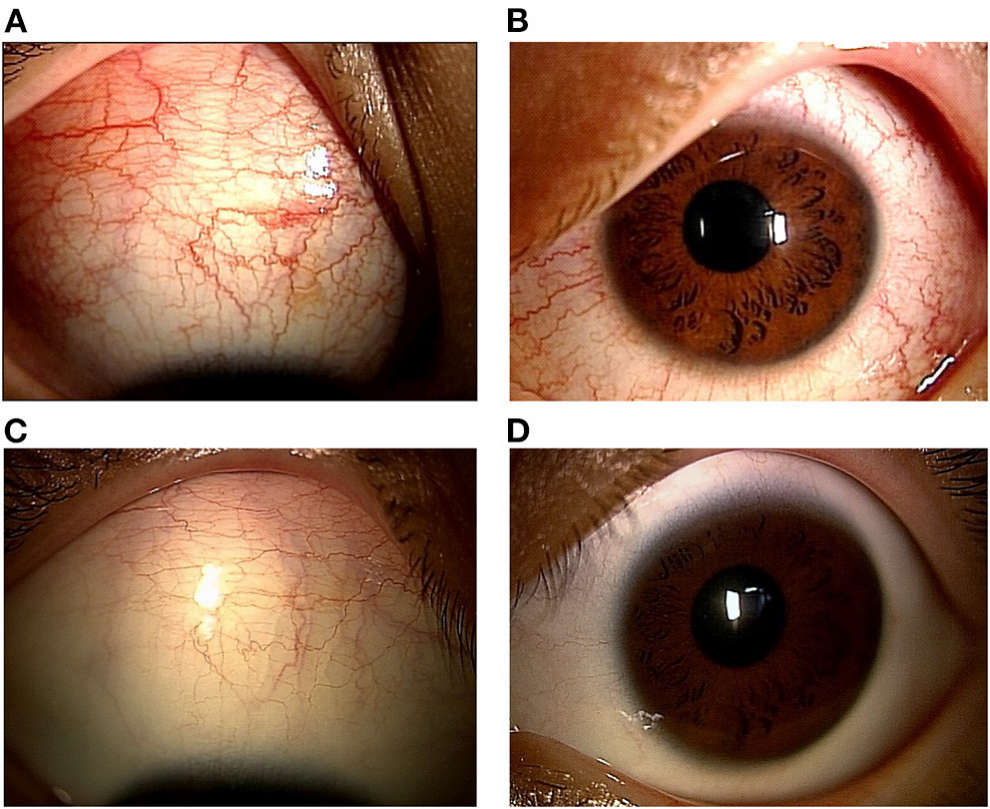
Ophthalmological slit-lamp examination before and after the intravenous immunoglobulin (IVIG) administration. Right **(A)** and left **(B)** slit-lamp photographs show bilateral conjunctival, episcleral and scleral njection, suggesting scleritis and concomitant conjunctivitis. Right **(C)** and left **(D)** slit-lamp photographs after IVIG administration, showing the dramatic improvement in their ocular findings.

**Figure 2:**
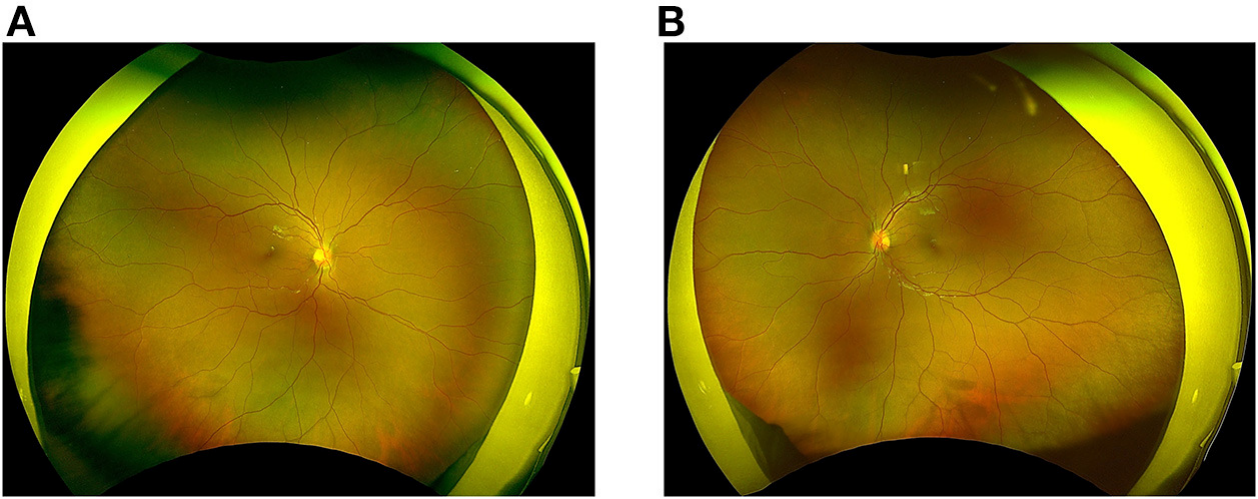
Fundus findings before the IVIG treatment. Right **(A)** and left **(B)** wide-field fundus photographs show no abnormal findings.

**Figure 3:**
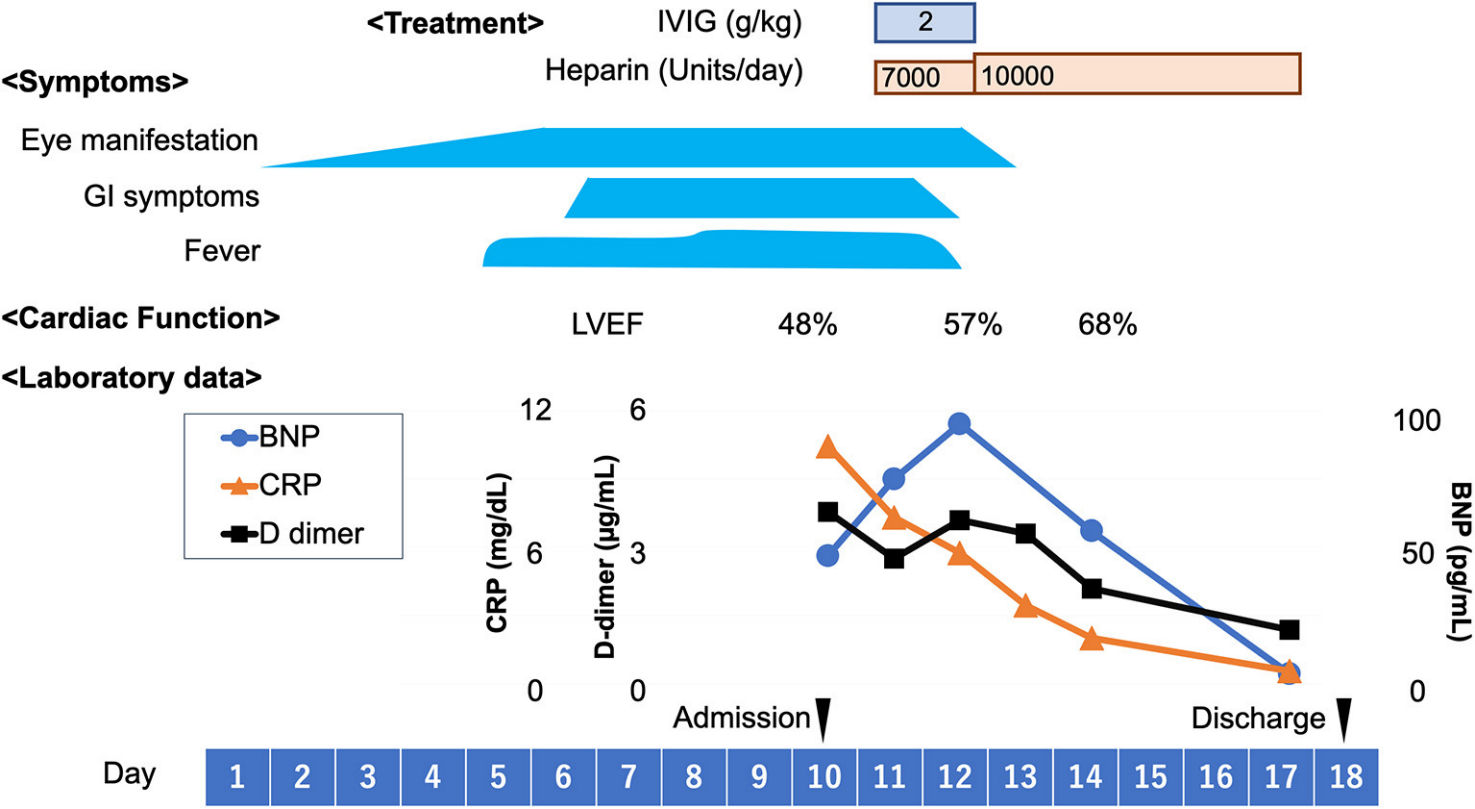
Clinical course. BNP, brain natriuretic peptide; CRP, C-reactive protein; GI, gastrointestinal; IVIG, intravenous immunoglobulin; LVEF, left ventricular ejection fraction.

A previously healthy 11-year-old Japanese boy was diagnosed with COVID-19 by nasopharyngeal polymerase chain reaction testing, attended with his parents. At the time, the delta variant was prevalent in Japan. The patient's only symptom was mild fever for 2 days. Five weeks later (day 1), he experienced bilateral eye pain that disturbed his sleep, and photophobia. On day 5, he developed a fever of 38.5°C, bilateral red eyes, and general malaise, followed by abdominal pain, frequent vomiting, and watery diarrhea on day 6. During this period, he visited an ophthalmology clinic several times, and was diagnosed with severe anterior scleritis and conjunctivitis of unknown origin based on the slit-lamp examination; the examination showed microvascular dilatation in various layers, including not only in the conjunctiva, but also in the episclera and deep sclera. Topical steroids (0.1% fluorometholone) did not improve the ocular symptoms. Subsequently, the patient presented with a high fever of 40°C and his general condition deteriorated, which led to his admission in our hospital on day 10. His vital signs were stable, with a body temperature of 37.7°C, respiratory rate of 16/min, heart rate of 117/min, blood pressure of 121/70 mmHg, and SpO_2_ of 98% on ambient air. Physical examination revealed cervical lymphadenopathy, abdominal tenderness, hypoactive bowel sounds, and rashes (dime-size annular plaques) on the inner thigh. Ophthalmological examination revealed anterior scleritis suggested by diffuse episcleral and deep scleral injection with microvascular dilatation, as well as bilateral conjunctivitis (Figures 1A,B). He did not show any discharges or matting of eyelashes. No uveitis, vitritis, or retinitis was observed. The visual acuity of the patient was normal. The fundal examination findings were unremarkable (Figure 2). Ocular ultrasonography was not performed. The film array showed no evidence of active viral infections, including those caused by SARS-CoV-2. Table 1 shows the laboratory findings. The patient presented a normal range of leukocytes with decreased lymphocyte count (600/μL), hyperinflammatory state (C-reactive protein of 10.5 mg/dL, procalcitonin of 0.86 ng/dL, serum amyloid A of 1,219 μg/mL, ferritin of 252 ng/dL, and lactase dehydrogenase of 495 U/L), hypercoagulopathy (FDP of 12.1 μg/mL and D-dimer of 3.8 μg/mL), and elevated markers of cardiac stress/damage (brain natriuretic peptide of 47 pg/mL, N-terminal pro-brain natriuretic peptide of 704 pg/mL, and troponin T of 0.02 ng/mL). The patient also showed liver dysfunction (alanine aminotransferase of 213 U/L, and aspartate aminotransferase of 385 U/L), and dehydration with hyponatremia and hypoalbuminemia. Chest radiography and electrocardiography findings were unremarkable. Echocardiography demonstrated decreased cardiac function with a left ventricular ejection fraction (LVEF) of 48% (using a modified Simpson method). Although the patient showed some features compatible with KD, he was diagnosed as having MIS-C based on the definition by the Centers for Disease Control and Prevention and the World Health Organization (3, 4). Following intravenous immunoglobulin (2 g/kg) administration and heparinization on day 11, his fever subsided by the next day (day 12). Notably, the ocular manifestations improved dramatically after intravenous immunoglobulin administration, without additional topical steroids (Figures 1C,D). Serial echocardiography demonstrated gradual improvement in LVEF, and LVEF improved to normal by day 14. The patient's coronary arteries were normal. The patient was discharged on day 18 and had uneventful cardiac and ocular manifestations 6 months after discharge (Figure 3).

**Table 1:** Laboratory test at admission.

**[Blood cell count]**			**[Film array]**	
White blood cell	9,800	/μL	**Viruses**	
Neutrophil	85.7	%	Adenovirus	N/D
Lymphocyte	0.1	%	Coronavirus 229E	N/D
Red blood cell	447 ×10^4^	/μL	Coronavirus HKU1	N/D
Hemoglobin	12.8	g/dL	Coronavirus NL63	N/D
Platelets count	30.8 ×10^4^	/μL	Coronavirus OC43	N/D
**[Coagulation test]**			SARS-CoV-2	N/D
FDP	12.1	μg/mL	Human metapneumovirus	N/D
Fibrinogen	739	mg/dL	Human Rhinovirus/Enterovirus	N/D
D-dimer	3.8	μg/mL	Influenza A, B	N/D
**[Blood biochemistry]**			Parainfluenza Virus 1, 2, 3, 4	N/D
CRP	10.5	mg/dL	Respiratory Syncytial Virus	N/D
Total Protein	7.6	g/dL	**Bacteria**	
Albumin	3	g/dL	*Bordetella parapertussis*	N/D
BUN	24	mg/dL	*Bordetella pertussis*	N/D
Creatinine	0.64	mg/dL	*Chlamydia pneumoniae*	N/D
Creatinine kinase	67	U/L	*Mycoplasma pneumoniae*	N/D
AST	385	U/L		
ALT	213	U/L		
LDH	495	U/L		
Sodium	135	mmol/L		
Potassium	4	mmol/L		
Chloride	99	mmol/L		
Procalcitonin	0.86	ng/dL		
Ferritin	252	ng/dL		
Serum Amyloid A	1,219	μg/mL		
NT-pBNP	704	pg/mL		
BNP	47	pg/mL		
Troponin T	0.02	ng/mL		

*ALT, alanine aminotransferase; AST, aspartate aminotransferase; BNP, brain natriuretic peptide; BUN, blood urea nitrogen; CRP, C-reactive protein; FDP, fibrinogen degradation products; LDH, lactase dehydrogenase; NT-pBNP, N-terminal pro-brain natriuretic peptide*.

## Discussion

MIS-C is defined as an individual aged less than 21 years who presents fever, laboratory evidence of inflammation, multisystem organ involvement (at least in 2 organ systems, such as ocular, dermatologic, gastrointestinal, and cardiac manifestations) and laboratory confirmed SARS-CoV-2 infection or epidemiologic link to a person with COVID-19 (3, 4). Therefore, MIS-C can present with various clinical features. While the literature is limited regarding ocular reports associated with MIS-C, anterior uveitis has recently been reported in addition to conjunctivitis (5–9). The present case was unique for the following two reasons: 1) the patient showed severe ocular manifestations as the primary symptom of MIS-C prior to other findings, such as fever or gastrointestinal manifestations, which could lead to delay in diagnosis, and 2) he suffered anterior scleritis, an unusual and severe form of inflammatory eye disease, rather than conjunctivitis. In addition, his detailed ocular examination provided us with clinical clues regarding the possible pathogenesis of MIS-C compared with KD.

To our knowledge, this is the first report of severe ocular manifestation as the primary symptom of MIS-C. Recently, several reports have demonstrated symptomatic anterior uveitis in a subset of patients with MIS-C (5–9). Notably, ocular symptoms associated with uveitis, such as blurring of vision or photophobia, became apparent at the subacute stage of disease, during or after the treatment of other MIS-C-related organ dysfunctions, such as myocarditis. The present case is unique in that the patient's severe ocular manifestation preceded other symptoms, such as fever, which could result in a delay in the diagnosis of MIS-C. We therefore recommend that pediatricians and physicians should keep in mind that severe ocular manifestations could be the first symptom of MIS-C, and MIS-C should be considered for a differential diagnosis, especially in COVID-19-prevalent areas.

The patient in the present case suffered from anterior scleritis as well as conjunctivitis. Ocular ultrasonography was not performed, and concurrent posterior scleritis was not fully ruled out. Since he first complained of bilateral eye pain and photophobia, followed by bilateral red eyes, scleritis could have preceded conjunctivitis. Scleritis is an inflammation in the episcleral and scleral tissues in both superficial and deep episcleral vessels, and can be vision-threatening. It is often associated with an underlying systemic disease (in up to 50% of patients), suggesting that the immune system may play a role in the pathogenesis of scleritis (10). To date, only one report has described bilateral anterior scleritis concomitant with anterior uveitis. In this report a 17-year-old boy with MIS-C complained of bilateral red eyes, eye pain, photophobia, and blurring of vision, with subsequent fever that occurred 1-week after post-admission, and after a 3-day course of intravenous methylprednisolone and tocilizumab administration (6). His eye condition required additional oral prednisolone and eye drops (0.1% dexamethasone) for up to a 6-week period. In contrast, in the MIS-C with scleritis case presented here, intravenous immunoglobulin was administered for MIS-C-related organ dysfunctions including fever, gastrointestinal symptoms, myocardial dysfunction and ocular symptoms, as the patient had been refractory to a steroid eye drop before admission. Fortunately, intravenous immunoglobulin administration alone immediately relieved the patient's eye symptoms together with other symptoms. He showed no ongoing inflammation thereafter. The precise mechanism is unknown why the immunoglobulin was effective for scleritis in the present case with MIS-C, as therapeutic options usually include steroid and immunosuppressive drugs for non-infectious scleritis (10). However, it is reasonable to consider immunoglobulin administration as a first step therapeutic option if the patient with MIS-C show severe ocular manifestation refractory to a steroid eye drop. Moreover, a thorough ophthalmological examination should be performed to avoid misdiagnosing the potentially vision-threatening condition of scleritis/uveitis as common conjunctivitis.

To some extent MIS-C mimics KD, and 40% of MIS-C cases meet the criteria for either complete or incomplete KD (2). Compared with KD, MIS-C more often presents with gastrointestinal (92%) and cardiovascular (80%) symptoms, both of which were seen in the present case (2). Considering eye manifestations, both MIS-C and KD show similar ocular inflammation in the form of conjunctivitis and anterior uveitis (11, 12). The fundal findings in KD have not been fully examined in the currently available English literature, probably because of technical problems with examining infants/young children (usually younger than MIS-C patients). Posterior involvement in KD is rare and is usually limited to case reports (13). Similarly, fundus findings in MIS-C are scarce in the literature, but some reports have demonstrated normal findings, as described in this case (6, 7, 9). Considering that both MIS-C and KD mainly involve the anterior segment of the eyes, we speculate that MIS-C and KD have a similar pathogenesis. To prove this hypothesis, more information using multimodalities, such as histopathological examinations, is required.

Coronary arterial abnormalities were unique findings in both MIS-C and KD groups. In patients with KD, they may determine the long-term morbidity and mortality in 20–25% of untreated cases; therefore, early diagnosis and treatment are essential to prevent cardiac complications (14). When examining the eyes for small vessel changes, we could predict the condition of the coronary arteries in patients with KD. Lim et al. (15) demonstrated a significant association between the diameter of coronary arteries and retinal arteriolar geometric changes in 11 children with new-onset of KD and with a median age of 5.9 years. Similarly, a subset of patients with MIS-C present with coronary complications, although the precise underlying mechanism is unclear. Our future studies will evaluate the vascular condition, including that of the coronary arteries, through the eyes using noninvasive techniques; this research could lead to a deeper understanding of the pathophysiology of MIS-C. The relatively older-age-onset of MIS-C compared to KD would be advantageous for such ophthalmologic examinations.

In conclusion, MIS-C presents with a wide variety of clinical manifestations, with the present case showing severe ocular manifestations in the form of anterior scleritis as the primary MIS-C symptom. In addition, ocular scleritis could be sight-threatening, and therefore it may require specific treatment in MIS-C cases such as intravenous immunoglobulin. Detailed ocular examinations by ophthalmologists could refine and guarantee the diagnosis of MIS-C, and could possibly help in further clarifying the MIS-C pathophysiology.

## Data Availability Statement

The raw data supporting the conclusions of this article will be made available by the authors, without undue reservation.

## Ethics Statement

Ethical review and approval was not required for the study on human participants in accordance with the local legislation and institutional requirements. Written informed consent to participate in this study was provided by the participants' legal guardian/next of kin. Written informed consent was obtained from the minor(s)' legal guardian/next of kin for the publication of any potentially identifiable images or data included in this article.

## Author Contributions

DM, DT, YK, YM, MH, and MS managed the patient, contributed to the conception of the study, and drafted the manuscript. YK and SM reviewed the manuscript from the ophthalmological perspective. TY critically reviewed the manuscript. All authors read and approved the final manuscript.

## Conflict of Interest

The authors declare that the research was conducted in the absence of any commercial or financial relationships that could be construed as a potential conflict of interest.

## Publisher's Note

All claims expressed in this article are solely those of the authors and do not necessarily represent those of their affiliated organizations, or those of the publisher, the editors and the reviewers. Any product that may be evaluated in this article, or claim that may be made by its manufacturer, is not guaranteed or endorsed by the publisher.
